# Genome-Wide Identification and Expression Profiling of the SPL Gene Family in *Musa acuminata*: Insights into Their Response to Drought Stress and *Serendipita indica* Inoculation

**DOI:** 10.3390/plants15091386

**Published:** 2026-04-30

**Authors:** Muniba Shafiq, Fengjie Yang, Zilu Yang, Ning Tong, Bowen Zhang, Dan Li, Muhammad Awais, Hafiz Muhammad Usman, Yuling Lin, Xu XuHan, Zhongxiong Lai

**Affiliations:** 1 Institute of Horticultural Biotechnology, Fujian Agriculture and Forestry University, Fuzhou 350002, China; munibashafiq@fafu.edu.cn (M.S.); fengjieyang1001@163.com (F.Y.); 17805953105@163.com (N.T.); bowenzhang120498@163.com (B.Z.); leed6335@126.com (D.L.); awais9518@gmail.com (M.A.); muhammadusman@fafu.edu.cn (H.M.U.); buliang84@163.com (Y.L.); 2 College of Horticulture, Fujian Agriculture and Forestry University, Fuzhou 350002, China

**Keywords:** banana, SPL, *miR156a*, drought stress, *Serendipita indica* (*Piriformospora indica*)

## Abstract

Banana productivity is severely limited by drought, yet the molecular basis of drought adaptation and endophyte-mediated stress alleviation remains poorly understood. Here, we performed a genome-wide analysis of the SQUAMOSA promoter-binding protein-like (SPL) transcription factor family in *Musa acuminata* and examined their transcriptional responses to drought stress and *Serendipita indica* inoculation. We identified 38 *MaSPL* genes, all encoding proteins with the conserved SBP domain and predicted nuclear localization. Phylogenetic, motif, gene structure, and collinearity analyses indicated that *MaSPL* genes are evolutionarily conserved, unevenly distributed across chromosomes, and expanded primarily through segmental duplication under purifying selection. Promoter analysis showed several *cis*-acting elements and transcription factor binding sites related to light, phytohormone, and stress signaling. Ten *MaSPL* genes were predicted as putative targets of miR156. qRT-PCR analysis showed that drought stress markedly downregulated the tested *MaSPL* genes, whereas *miR156a* expression increased, supporting an inverse regulatory relationship. Under drought, *S. indica* inoculation enhanced expression of most tested *MaSPLs*, restoring transcript accumulation while reducing *miR156a* to near-basal levels. Notable responses were observed in members of the *MaSPL2*, *MaSPL9*, and *MaSPL13*, respectively. *S. indica* improves drought tolerance by enhancing antioxidant defenses, reducing oxidative stress, and preserving photosynthetic and osmotic stability. Taken together, our results demonstrate that *S. indica* confers drought resilience in banana by counteracting drought-induced repression of *MaSPL* genes via the miR156–SPL module and by strengthening key physiological defense mechanisms.

## 1. Introduction

Banana (*Musa* spp.) is among the most economically and nutritionally significant fruit crops grown globally. The Food and Agriculture Organization (FAO) recognized it as the fourth most important staple food in developing nations [1]. China ranks as the second-largest banana producer globally. In 2022, the country’s banana cultivation area reached approximately 505,000 hectares, yielding a total production of 11.77 million tons [2]. Due to its shallow root system and high water demand, it is particularly sensitive to water shortages. However, banana productivity is significantly constrained by both biotic and abiotic stresses, with drought emerging as a major limiting factor due to the increasing impacts of climate change. As an economically significant fruit crop cultivated predominantly in tropical and subtropical regions, banana is especially sensitive to water deficit conditions, which can severely impair yield. However, despite its global importance as both a food and cash crop, the molecular processes involved in its stress response remain relatively underexplored compared with those of other major crops.

In parallel with genetic and transcriptome-based strategies, beneficial root-associated endophytes are increasingly recognized as practical tools to improve drought resilience in horticultural crops [3]. The root-colonizing basidiomycete *Serendipita indica* (syn. *Piriformospora indica*) establishes a mutualistic association with a wide host range and has been reported to enhance plant performance under water deficit by improving root system function, nutrient acquisition, osmotic adjustment, and antioxidant capacity, while also modulating stress-related hormone and transcriptional networks [4]. Consequently, *S. indica* mediated stress mitigation provides a biologically meaningful context to investigate whether drought-responsive transcription factor families, including SPL/SBP, are transcriptionally reprogrammed during water stress and endophyte colonization.

Transcription factors (TFs) are sequence-specific DNA-binding proteins that regulate gene expression by activating or repressing downstream target genes [5]. They regulate complex mechanisms which are involved in abiotic stress and plant development [6]. Major TF families, including MYB, NAC, WRKY, AP2/ERF, and SBP/SPL, have been widely reported to function in stress adaptation.

The SQUAMOSA promoter-binding protein-like (SPL) family represents a plant-specific group of transcription factors characterized by a highly conserved SBP domain. The SBP domain contains three essential motifs: two zinc-binding sites, Cys–Cys–Cys–His (Zn1) and Cys–Cys–His–Cys (Zn2), as well as a nuclear localization signal (NLS) at the C-terminal region, which overlaps with second zinc-finger motif, ensuring proper nuclear targeting [7,8]. The nuclear localization signal (NLS) facilitates the transport of the SPL protein into the nucleus, where it regulates the transcription of target genes. As a regulatory protein, SPL modulates gene expression by either activating or repressing target genes through specific recognition and binding to the conserved GTAC *cis*-element present in their promoter regions [9]. SPL genes were initially identified through the characterization of *SBP1* and *SBP2* in *Antirrhinum majus* [10]. Many SPL gene families have been studied in several plant species, like *Arabidopsis thaliana* [11,12], *Oryza sativa* [13], *Zea mays* [14], and *Triticum aestivum* [15]. Functional characterization of SPL genes in these plants indicates that they play key regulatory roles in multiple developmental and physiological processes, including flower and fruit development, stress response, leaf development, phase transition, plant architecture, and petal and sepal development.

SPL genes constitute an important regulatory network involved in plant responses to abiotic stress. In rice, *OsSPL10* has been reported to regulate tolerance to environmental stresses, including drought and salinity [16], whereas *OsSPL3* is associated with crown root development [17]. Members of the SPL family also contribute to reproductive development. For example, *TaSPL13-2B* has been shown to control floret development in wheat [18], while overexpression of *MsSPL20* in alfalfa results in delayed flowering. In addition, SPL genes influence stress adaptation in several species. In Arabidopsis, *AtSPL9* enhances freezing tolerance through activation of *CBF2* expression [19]. In alfalfa, *SPL9-RNAi* lines exhibit improved drought tolerance, whereas miR156-overexpressing plants and *SPL13-RNAi* lines display greater tolerance to heat stress [20,21]. Conversely, *OsSPL10* has been found to negatively regulate salt tolerance in rice [22].

MicroRNAs (miRNAs) are endogenous, small non-coding RNAs (approximately 20–24 nucleotides) that regulate gene expression primarily by mRNA cleavage or translational inhibition. Certain members of the SPL gene family are targeted by miR156/miR157, which plays a crucial role in regulating various phases of plant growth [23]. Numerous studies have demonstrated that either overexpressing or knocking down miRNAs, including miR156 [24,25], miR166 [26], miR319 [27], and miR408 [28], can increase plant stress resistance to biotic or abiotic stressors. Overexpression of miR156a reduces apples’ ability to withstand salt [29]. Abiotic stress resistance can be improved by miR166 knockdown [30]. Switchgrass’s salt tolerance is positively regulated by miR319, as evidenced by the overexpression of MIR319b and miR319 in the target simulated form MIM319 [31]. By altering the shape of their leaves, Os-miR408 transgenic plants can become more drought resistant. Among them, miR156 is one of the most evolutionarily conserved and functionally significant in plants. MiR156 specifically targets multiple SPL genes, thereby acting as a key regulator for phase transitions [32], stress responses [33], and flower development [34]. This miR156–SPL regulatory module has been reported to enhance drought tolerance by modulating stomatal behavior, ROS detoxification, and root system architecture. According to a recent study, the silencing of SPL gene by miR156 enhanced resistance to drought stress and encouraged Abscisic acid sensitivity and leaf gas exchange [35,36]. By suppressing *SPL13* expression, miR156 was demonstrated to increase drought resistance in alfalfa; in the same study, overexpression of miR156 resulted in increased drought resistance [24].

A previous study [37] identified 56 putative SPL genes in *M. acuminata* and mainly associated them with fruit development and ripening; however, little is known about their contribution to abiotic stress adaptation, particularly drought tolerance and its modulation by beneficial fungal symbionts. A total of 38 putative SPL gene were identified in the banana genome in this study. Furthermore, the transcriptional responses of *MaSPL* genes under combined drought stress and *S. indica* treatment were analyzed by qRT-PCR. Despite growing evidence for both SPL mediated stress regulation and *S. indica*–induced drought tolerance, it remains unclear whether endophyte colonization reshapes the miR156–SPL regulatory network in banana under water deficit conditions.

## 2. Materials and Methods

### 2.1. Plant Material and Treatment

**Figure 1:**
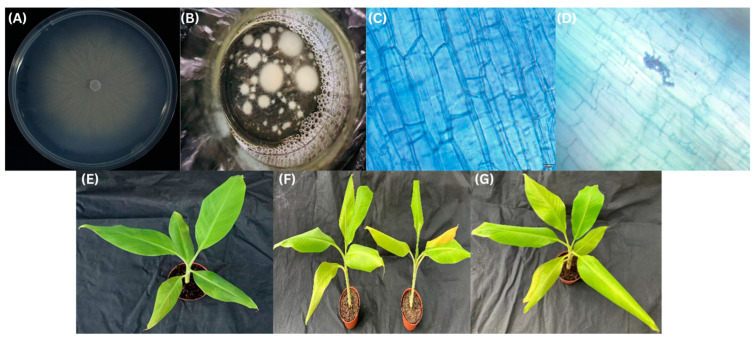
Phenotypic and microscopic observations of ‘Brazilian’ banana seedlings under drought stress and *S. indica* inoculation. (**A**) Growth of *S. indica* on potato dextrose agar (PDA), showing radial mycelial expansion from the inoculation point. The mycelium initially appeared white and gradually turned pale yellow as it matured, forming a yellowish-white powdery spore mass. (**B**) *S. indica* spore suspension prepared in potato dextrose broth (PDB). (**C**) Root colonization by *S. indica*. (**D**) Root sample without fungal colonization. (**E**) Ck: watering + non-inoculation; (**F**) T1: drought + non-inoculation; (**G**) T2: drought + inoculation.

In vivo-grown ‘Brazilian’ (*M. acuminata* L., AAA group, cv. Brazilian) plantlets with uniform growth, approximately 18 cm in height and bearing 6–7 leaves, were transplanted into pots containing an autoclaved substrate of peat soil, vermiculite, and perlite (4:2:1). After autoclaving, the substrate was moistened with sterile distilled water, and all plantlets were watered once immediately after transplanting to ensure establishment. Subsequently, *S. indica* inoculation was performed, and drought treatment was initiated after inoculation according to the experimental design. There are three treatments: watering + non-inoculation (CK), drought + non-inoculation (T1), and drought + inoculation (T2); each treatment set up three replicates, and each replicated have 10 plants. The preparation of *S. indica* fermentation broth was carried out according to our lab’s optimized protocol [38]. After fungus growth on PDA plates outgrowing fresh fungus (Figure 1A) was cut approximately 0.6 mm in size with the help of micropipette tips put in 200 mL PDB for shaking at 37 °C for 3–4 days (Figure 1B), after colony increase dissolved in distilled water with 100 mL of fungus liquid and 200 mL distilled water. In total, 100 mL of diluted spore suspension was added to the soil of T2 plants every 3 days, poured five times in total, and watering was stopped after the last pouring.

After *S. indica* and banana seedlings grew together for 32 days, the roots of 10 banana plants were randomly collected, and the non-colonization and colonization status of *S. indica* in the roots were examined by trypan blue staining [39]. Drought stress was imposed using a drought–rewatering regime with slight modifications to the method of [40]. During treatment, plants were monitored daily for wilting symptoms, and pot weight was recorded to estimate substrate water content. Drought-stressed plants were allowed to dry until substrate water content decreased to 20–30% of saturated water content. When wilting symptoms persisted for three consecutive days without recovery, the plants were rewatered to 73–78% of saturated water content until the wilting symptoms disappeared. This drought rewatering procedure was repeated three times. Control plants were maintained under well-watered conditions at approximately 75% field water capacity. Leaf and root samples were collected, flash-frozen in liquid nitrogen, and stored at −80 °C after completion of the third drought rewatering cycle for subsequent analyses (Figure 1).

### 2.2. Measurement of Antioxidant Enzyme Activities

From each sample, 0.1 g was collected, rapidly frozen in liquid nitrogen, and finely powdered, followed by homogenization in the extraction buffer provided he commercial kits (Keming, Suzhou, China) using a pre-cooled mortar in an ice bath. The homogenate was centrifuged at 10,000× *g* for 10 min at 4 °C, and the supernatant was collected and kept on ice. Absorbance was determined using an ultraviolet-visible spectrophotometer (T6, Puxi, Beijing, China) and the activities of SOD, POD, CAT, MDA, and chlorophyll content were determined according to the kit instructions.

### 2.3. Identification and Characterization of MaSPL

The SPL gene family in banana were identified using publicly available genomic and protein databases. The whole-genome sequence of *M. acuminata_Pahang_v4* was retrieved from the Banana Genome Hub, while SPL protein sequences of *A. thaliana* were used as queries in TBtools-II version 2.080 searches to identify homologous family members in banana. The *Arabidopsis* SPL IDs were retrieved based on the conserved SPL Pfam domain (PF03110), and candidate banana SPL proteins were identified using a bidirectional BLAST approach [41]. Candidate sequences were further verified as members of the SPL gene family using the Genome.jp database. The retrieved sequences were regarded as putative SPL family members for further analysis. These retrieved sequences were further renamed on the basis of *A. thaliana* nomenclature. Their physicochemical properties, including amino acid length, molecular weight, isoelectric point, instability index, and GRAVY, were analyzed using the ExPASy ProtParam tool (http://web.expasy.org/protparam/, accessed on 20 April 2026) [42], and subcellular localization was predicted using the tool WoLF PSORT (https://wolfpsort.hgc.jp/, accessed on 20 April 2026). Phylogenetic analysis was conducted in MEGA 12 using the Neighbor-Joining method based on 73 SPL amino acid sequences from *M. acuminata*, *O. sativa*, and *A. thaliana*, with the Poisson model, complete deletion, and 1000 bootstrap replicates.

### 2.4. Gene Structure and Conserved Motif Analysis of MaSPL Genes

The exon–intron structure of the SPL gene family was determined using Gene Structure Display Server (GSDS, version 2.0). Genomic sequences of the SPL gene family were aligned with their corresponding cDNA sequences to map the exon–intron boundaries. The conserved protein domains in each SPL protein were predicted using NCBI’s Conserved Domain Database (CDD) [43]. The presences of specific motifs were analyzed trough online website MEME (Multiple Expectation Maximization for Motif Elicitation) [44]. The physical localization of these motifs along the protein sequences was recorded to highlight conserved domains essential for protein function.

### 2.5. Chromosomal Distribution and Collinearity Analysis

The chromosomal locations of the identified *MaSPL* genes were obtained from the banana genome annotation available in the Banana Genome Hub, and their distribution across chromosomes was visualized using TBtools-II version 2.080 [41]. Gene positions were recorded in a table and visualized using Circoletto, an online visualization tool based on Circos [45]. The distribution of SPL across chromosomes was analyzed to check for patterns, such as clustering of genes or uneven distribution. Chromosomes with the highest gene density were identified and further analyzed. To investigate the evolutionary relationship and collinearity of the *MaSPL*, a collinearity analysis was performed with SPL genes from Arabidopsis and rice as reference species. Collinearity was assessed by aligning the banana genes with corresponding orthologs in *Arabidopsis* and rice using MCScanX tool function in TBtools-II version 2.080 [46]. The identified SPL was examined for segmental and tandem duplications. Duplicated gene pairs were further analyzed for their Ka/Ks (nonsynonymous/synonymous substitution rate) ratios to assess the selective pressures acting on the gene family during evolution. A Ka/Ks value below 1 is generally interpreted as evidence of purifying selection, whereas a value above 1 is indicative of positive selection.

### 2.6. Promoter Analysis, and Transcription Factors Binding Sites of MaSPL Genes

The 2000-bp upstream promoter regions of the *MaSPLs* were extracted from the banana genomic sequences. Cis-acting elements in these promoter regions were identified using the PlantCARE database [47], which annotates regulatory elements involved in many biological processes, such as stress responses, light response, and hormone signaling (Supplementary Table S5). The identified cis-elements were classified into functional categories, including elements related to stress, light, and phytohormone responses, and were compared across the different banana species. The transcription factor binding sites within the promoter regions were predicted using the PlantTFDB online (http://planttfdb.cbi.pku.edu.cn/, accessed on 28 December 2026) platform with a significance threshold of *p* ≤ 1 × 10^−6^ [48]. The resulting data were subsequently compared, analyzed, and visualized using TBtools software [41].

### 2.7. MicroRNA Target Prediction in SPL Transcription Factors of MaSPL

To investigate potential post-transcriptional regulation of SPL transcription factors in *M. acuminata*, we performed a genome-wide prediction of miRNA target sites. The coding sequences (CDSs) of all identified SPL genes were used as queries in the psRNATarget server (accessed on 1 January 2026, at https://www.zhaolab.org/psRNATarget/) with defaulting parameters. Target prediction was based on sequence complementarity and target site accessibility, and stringent filtering was applied to retain only high-confidence interactions. Predicted miRNA binding sites were subsequently mapped to both coding and untranslated regions (UTRs) of the SPL gene models. Only miRNA–target pairs with high complementarity scores were considered for downstream analyses [49].

### 2.8. Transcriptome Sequencing and Differential Expression Analysis

Transcriptome sequencing and differential expression analysis were conducted using root samples collected from CK, T1, and T2 treatments. RNA was isolated from roots using the FastPure Universal Plant Total RNA Isolation Kit (Nanjing Vazyme Biotech Co., Ltd., Nanjing, China). Differentially expressed *MaSPL* genes and miR156a were identified from the root transcriptome and sRNAome dataset, and the corresponding log2 fold-change values are provided in (Tables S3 and S4). Selected *MaSPL* genes were further validated by qRT-PCR. For cDNA synthesis, total RNA was reverse-transcribed using the RevertAid Master Mix with DNase I treatment kit (Thermo Fisher Scientific, Shanghai, China). For miRNA expression analysis, total RNA was separately reverse-transcribed using the TransScript® miRNA First-Strand cDNA Synthesis SuperMix (TransGen Biotech, Beijing, China). RT-qPCR was performed with HRbio^TM^ qPCR SYBR Green Master Mix (No Rox) (He Rui, Fujian, China) for quantitative analysis of target genes. *CAC* was used as the internal reference gene for mRNA expression analysis, while *U6* used as the reference gene for miRNA quantification [50]. All primer sequences are listed in (Supplementary Table S6). The Roche LightCycler 480 system is used to amplify the relative expression level of miRNA and its target genes. Each sample was analyzed using three biological replicates, and mean values were used for further evaluation. Relative expression levels were determined according to the 2^−ΔΔCT^ method [51,52].

### 2.9. Statistical Analysis

Data processing, figure preparation, and statistical analyses were carried out using GraphPad Prism 8.02 software (GraphPad Software, San Diego, CA, USA) and Statistix 8.1 (Analytical Software, Tallahassee, FL, USA). All experiments were conducted with three biological replicates, and the results are presented as mean ± standard deviation (SD). Prior to analysis, data were tested for normality and homogeneity of variance to ensure the validity of parametric tests. Differences among treatments were evaluated using one-way analysis of variance (ANOVA), followed by the least significant difference (LSD) test for multiple comparisons. Statistical significance was determined at *p* < 0.05.

## 3. Results

### 3.1. Identification of SPL Genes in M. acuminata

A systematic genome-wide analysis of the *MaSPL* gene family was performed, resulting in the identification and characterization of 38 *MaSPL* genes. The predicted proteins varied considerably in size, with amino acid lengths ranging from 171 in (*MaSPL4a*) to 506 in *(MaSPL2f)* (Table 1). Similarly, the molecular weights of the encoded proteins differed substantially, with the highest value recorded for *MaSPL2f* at 56.488 kDa and the lowest for *MaSPL4a* at 18.981 kDa. The pI values ranged from 5.84 to 9.67 (*MaSPL13a and MaSPL5*), indicating that most of these are alkaline proteins, with an average value of 8.54. Based on the instability index (II), all *MaSPL* were classified as unstable proteins, as their instability coefficients exceeded 40. All 38 *MaSPLs* proteins were predicted to be hydrophilic because their grand average of hydropathicity (GRAVY) scores were minus, ranging from −0.352 *(MaSPL2e*) to −1.226 *(MaSPL5*) (Table 2). The conserved SBP domain were identified in all *MaSPL* proteins, and subcellular localization analysis predicted that each member is localized in the nucleus.

### 3.2. Phylogenetic Analysis of MaSPL Genes

**Figure 2:**
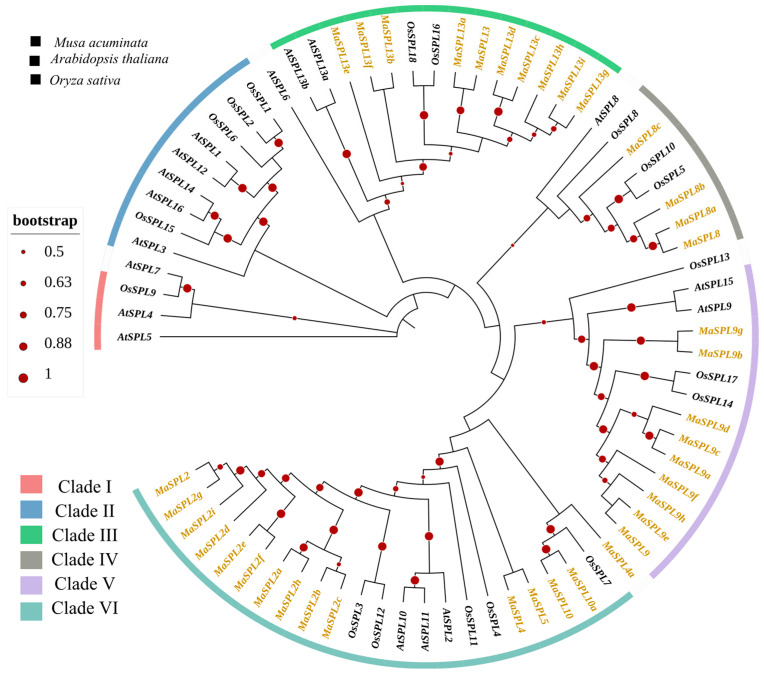
Maximum likelihood phylogenetic tree of SPL proteins from *M. acuminata*, *A. thaliana*, and *O. sativa*. SPL members were grouped into six distinct clades, Clades I–VI. Ma, *M. acuminata*; At, *Arabidopsis thaliana*; Os, *O. sativa*. Gene names shown in different font colors indicate different species: *MaSPL* genes are shown in golden/yellow font, while *AtSPL* and *OsSPL* genes are shown in black font. Red circles on the nodes indicate bootstrap support values.

To understand the phylogenetic relationships among SPL in other plants, we constructed a phylogenetic tree using multiple sequence alignments of SPL proteins from banana 38 *MaSPLs*, 18 *OsSPLs* from rice, and 17 *AtSPLs* from *Arabidopsis*; their gene Id and gene rename was mentioned in Table 1 and Supplementary Table S1, respectively. All these genes showed a conserved domain of SPL (Supplementary Figure S1). Thirty eight *MaSPL* members were classified into six major clades based on high-confidence bootstrap support values (Figure 2). Clade I included fourteen members of *MaSPL* clustered with *AtSPL10/11/2* and *OsSPL3/12/11/4/7* with 0.991 bootstrap values. Clade II showed all members of *MaSPL13* clustered with *AtSPL6/13/13a* and *OsSPL18/16* with the value of 0.925. For clade III, all members of *MaSPL8* clustered with *AtSPL8* and *OsSPL8/10/5*, with a bootstrap value of 0.836. Clade V included nine members of *MaSPL* clustered with *AtSPL9/15* and *OsSPL13/14/17* with a bootstrap value of 1 which indicates strong evolutionary conservation. Clades IV and VI did not show any direct relationship with banana. These findings showed that *MaSPL AtSPL* and *OsSPL* clustered together, suggesting that these proteins might have identical biological roles.

### 3.3. Characterization of Conserved Motifs and Structural Arrangement of MaSPL

**Figure 3:**
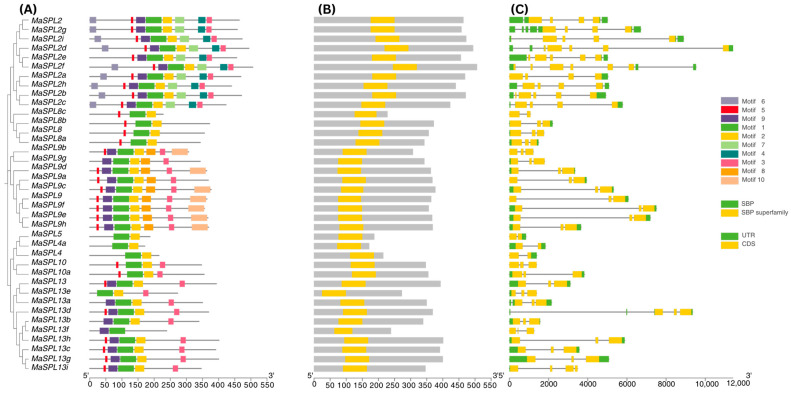
Phylogenetic tree, conserved motif distribution, gene structure, and exon/intron distribution analysis of *MaSPL* gene members in banana. (**A**) Phylogenetic tree was constructed based on *MaSPL* proteins and MEME-based motif prediction of *MaSPL* proteins; motifs are shown as colored blocks (motifs 1–10). (**B**) Each gray horizontal box represents the full length of an analyzed sequence, while the yellow boxes indicate the predicted SBP superfamily domain within each sequence. The scale at the bottom shows the relative sequence length and position from 5′ to 3 (**C**) Gene structure of *MaSPL* showing exons, and introns, indicated by yellow boxes and introns by black connecting lines.

The Tbtools software was used to predict motifs of *MaSPL* protein sequences. In all, 10 motifs, named motifs 1 to 10, were identified (Figure 3). The length of 10 identified motifs and consensus sequence were listed in (Supplementary Table S2). The lengths of those conserved motifs were between 08 (motif 05) to 50 amino acids (motif 01 and motif 10). Several motifs displayed distinct positional preferences within the sequences. Specifically, motifs 05 and 09 were consistently located near the beginning of the motif arrangement, whereas motifs 01 and 02 were typically found together. The number of the conserved motifs in each *MaSPL* varied from 2 to 08. All members of the *MaSPL2* proteins showed the same number of motifs, in which motif 8 and motif 10 were missing. All members of the *MaSPL8* protein showed motif 01, 02, and 05. *MaSPL9* members showed lack of motif 04, motif 06, and motif 07. *MaSPL5, MaSPL4, MaSPL4a, MaSPL10*, and *MaSPL10a* showed similar motif distribution. In general, we observed that genes belonging to the same subfamily clustered together and had similar structural compositions, which was aligned with the phylogenetic tree-based classification.

We also found that all *MaSPL* genes contained the SBP conserved domain. The findings demonstrated the number of exons, ranging from 02 to 06. *MaSPL2f* exhibited the highest number of exons (06), whereas *MaSPL8c*, *MaSPL5, MaSPL4*, and *MaSPL4a* have only 02 exons. Most of the *MaSPL9* and *MaSPL13* family members have 03 exons. The *MaSPL* and *A. thaliana SPL* gene structures were analyzed and proved quite similar to *AtSPLs*. In *Arabidopsis*, *AtSPL4* and *AtSPL5* had 02 exons; *AtSPL8, AtSPL9,* and *AtSPL13a/b* had 03 exons; while, *AtSPL2* had 04 exons, respectively (Supplementary Figure S2). This finding demonstrates that different *MaSPL* orthologs have distinct exon–intron architectures and were similar to *Arabidopsis* orthologs.

### 3.4. Chromosome Localization and Collinearity Analysis of MaSPL

**Figure 4:**
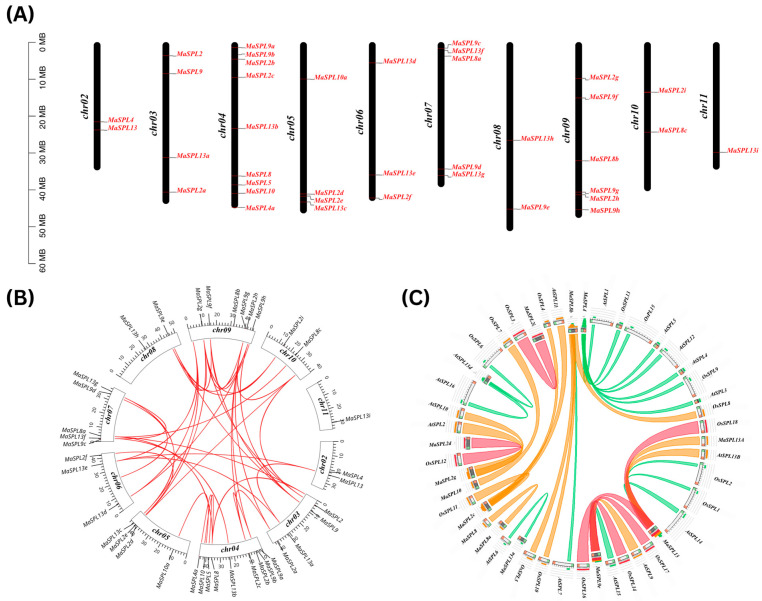
Chromosomal distribution and collinear analysis of *MaSPL* gene members. (**A**) The left-hand scale indicates chromosome length, Chromosomes are shown as black bars, with the corresponding chromosome numbers labeled to the left bar. (**B**) Circos visualization of segmental duplication relationships among *MaSPL* genes showed in red lines in the banana genome. (**C**) Circos analysis showing collinearity of SPL proteins among banana, rice, and Arabidopsis. Colored links indicate collinear relationships across the three species. The links are color-coded according to the ‘score/max’ ratio: green (≤0.50), orange (≤0.75), and red (>0.75).

Chromosomal distribution analysis revealed that SPL genes are mapped across different chromosomes, as determined using the latest version of the banana genome database. The 38 *MaSPLs* were unevenly distributed across ten chromosomes (02–11) (Figure 4A). Chr04 harbored the highest number of *MaSPL* (09 genes, approximately 23.68%), followed by Chr09 and Chr07, which contained six and five genes, respectively (approximately 15.78% and 13.15%). Chr03 and Chr05 each contained four *MaSPL* (approximately 10.5%), whereas Chr11 showed the least number of *MaSPL* (01 gene, approximately 2.63%). The chromosomal positioning of *MaSPL* genes appeared random, and no significant association was observed between their distribution and corresponding features (Figure 4B). Collinearity analysis further indicated that segmental duplication was the predominant mechanism contributing to *MaSPL* family expansion, while no tandem duplication events were detected under the applied distance criterion. Consistently, Ka/Ks analysis showed that most duplicated gene pairs were in purifying (negative) selection (Ka/Ks < 1), with only a few pairs exhibiting signatures of positive selection (Ka/Ks > 1), suggesting overall evolutionary conservation with limited adaptive divergence.

To explore evolutionary conservation of the SPL family, comparative synteny analysis was performed among *MaSPL*, *AtSPL*, and *OsSPL*. The Circos plot (Figure 4C) revealed multiple collinear links between banana *SPL* and their Arabidopsis/rice counterparts, indicating partial conservation of SPL loci across monocots and dicots together with lineage-specific diversification. Notably, *MaSPL4* showed extensive synteny with several *AtSPLs* (*AtSPL1/3/4/5/12*) and *OsSPLs* (*OsSPL9/13/15*), suggesting it represents an ancient, conserved SPL lineage. *MaSPL13* displayed strong collinearity with *AtSPL13A/13B/14* and *OsSPL1/2/6/18*, while *MaSPL9e* was syntenic with *AtSPL9* and *OsSPL14/15/17*, supporting evolutionary stability of these genes. In contrast, *MaSPL13a* linked only to *AtSPL6*, whereas *MaSPL8a* and *MaSPL10a* were exclusively connected to *OsSPL8* and *OsSPL10*, respectively, implying monocot-biased retention/divergence. Additional conserved links were observed for *MaSPL2g* (*AtSPL2/AtSPL10*) and *MaSPL2c* (*AtSPL11 and OsSPL4/11*), suggesting reservation of key regulatory functions across lineages.

### 3.5. Cis-Regulatory Element and Transcription Factor Binding Site Analysis of MaSPL Promoters

**Figure 5:**
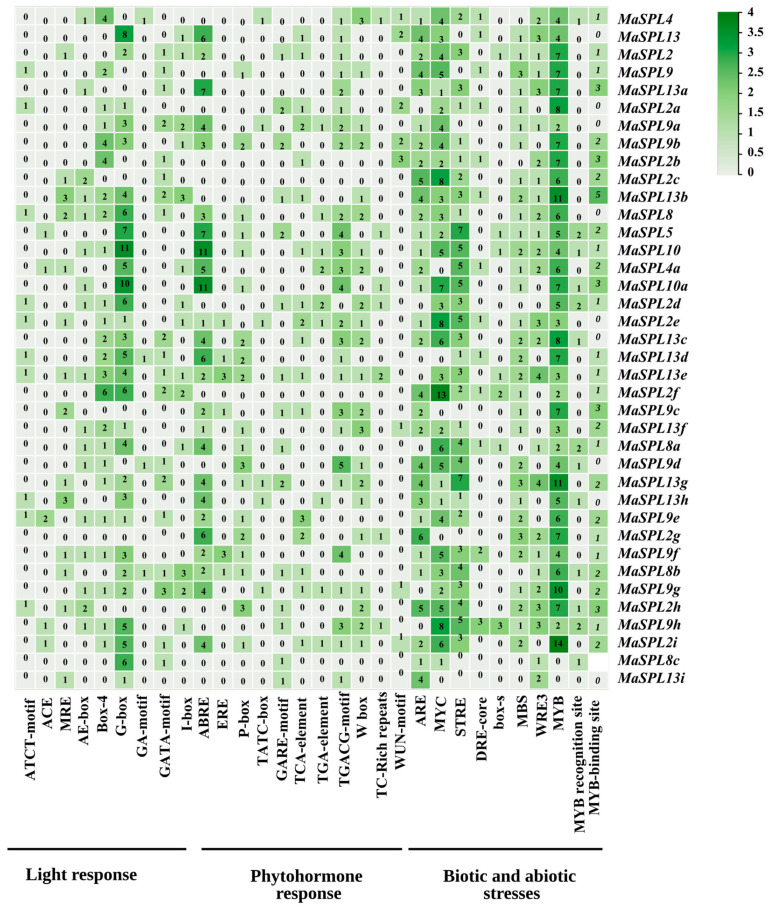
We analyzed the 2kb promoter sequence upstream of *MaSPL* genes in banana to identify cis-acting elements. Numbers within each cell represents the frequency of each *cis*-element values greater than 4 (highlighted in the heatmap) denote strongly enriched elements potentially.

The 2 kb upstream promoter sequences of *MaSPL* genes were retrieved from the Banana Genome Database. Analysis using PlantCARE identified a total of 1306 cis-regulatory elements within the promoter regions of these genes (Figure 5). These elements were broadly categorized into three functional groups: light-responsive, phytohormone-responsive, and stress-responsive elements, consistent with previous reports. Among them, 272 elements were associated with light responsiveness, including ACE, AE-box, Box 4, G-box, and GATA-motif; 391 elements were related to phytohormone responsiveness, including ABRE, ERE, P-box, TATC-box, and TGACG-motif; and 777 elements were involved in stress responsiveness, including MYC, MYB, DRE core, WRE3, STRE, WUN-motif, ARE, and Box S. Within the stress-responsive category, MYB elements were the most abundant, accounting for 33.28% of the total, whereas in the phytohormone-responsive category, the ABRE motif was the predominant element (27.36%), followed by ARE (19.94%). Among the light-responsive elements, the G-box was the most prevalent (44.11%), followed by Box 4 (16.91%). Notably, the distribution of cis-acting regulatory elements varied considerably among different *MaSPL* genes. For instance, *MaSPL10* contained a relatively higher proportion of light-responsive cis-elements. Among the stress-responsive elements, MYB was most abundant in *MaSPL2i*, followed by *MaSPL13b* and *MaSPL13g,* whereas MYC was highly represented in *MaSPL2f*.

Additionally, the regulatory relationship between *MaSPL* transcription factors were predicted and analyzed (Supplementary Figure S3). In total, binding sites for transcription factors of 25 families were identified in *MaSPL* promoters, including WOX, NAC, HSF, GATA, Dof, SBP, BBR-BPC, Dof, MIKC_MADS, AP2, bHLH, C2H2, B3, CPP, BES1, bZIP, G2-like, C3H, MYB, TCP, Trihelix, and WRKY. Among these, BBR-BPC binding sites were the most abundant (1455 sites) followed by MIKC_MADS (1051 sites), and the fewest binding sites were observed in E2F/DP, respectively. These observations imply that *MaSPL* genes may play important roles in multiple physiological and developmental processes, as well as in adaptation to abiotic stresses, with different transcription factors exerting specific regulatory influences on their expression.

### 3.6. Analysis of Predicted miR156 Target Sites in MaSPL Genes

**Figure 6:**
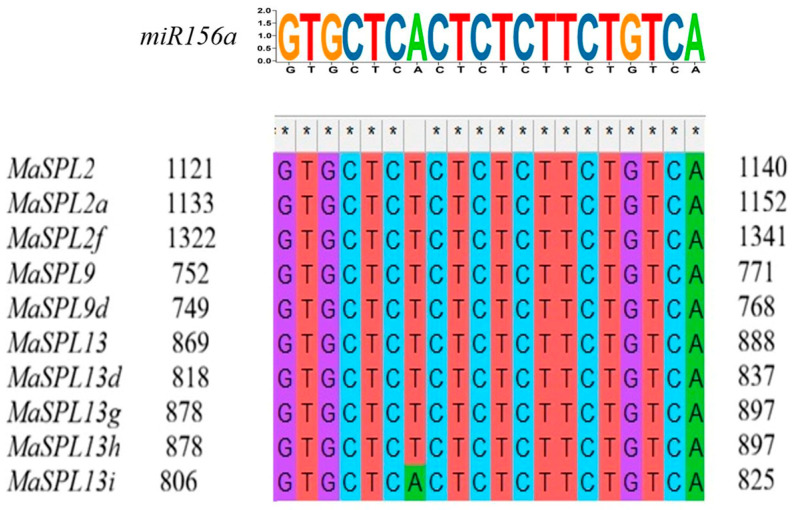
To identify potential *miR156a* target sites within *MaSPL* genes, coding sequences were analyzed using the psRNATarget tool (accessed on 1 January 2026, at https://www.zhaolab.org/psRNATarget/). The mature miR156 sequence used for this analysis was retrieved from the miRBase database (accessed on 1 January 2026, at https://www.mirbase.org/), Asterisks (*) denote nucleotide mismatches between *MaSPL* and *miR156a*; in plants, these mismatches are expected features of functional miRNA target sites and do not prevent binding.

To investigate the potential post-transcriptional regulation of *MaSPL* genes by miR156, we performed a multiple sequence alignment to identify *miR156a* target sites within their coding sequences (Figure 6). This analysis revealed complementary target sites in ten *MaSPL* genes, suggesting that these family members are putative targets of miR156-mediated regulation. Specifically, target sites were identified within the coding regions of *MaSPL2, MaSPL2a*, and *MaSPL2f* (belonging to clade VI), *MaSPL9* and *MaSPL9d* (clade V), as well as *MaSPL13, MaSPL13d, MaSPL13g, MaSPL13h*, and *MaSPL13i* (clade III). The presence of these sites across diverse phylogenetic clades indicates that post-transcriptional control of SPL genes by miR156 is a broadly conserved mechanism in plants.

### 3.7. Expression Patterns of MaSPL Genes Across Different Tissues

**Figure 7:**
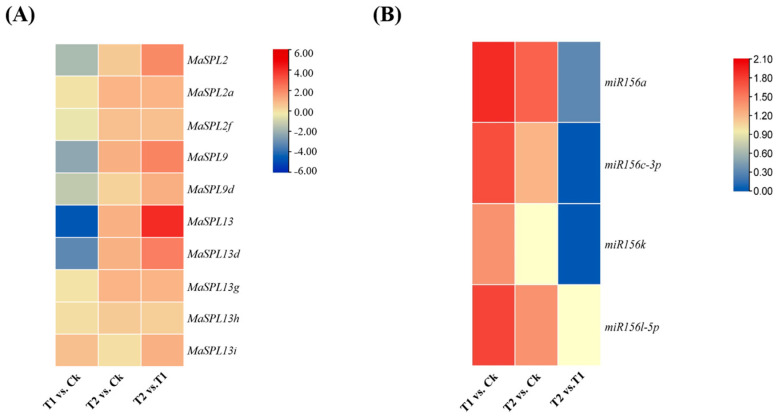
Heatmap showing the expression patterns of *MaSPL* and *miR156a/c-3p/l-5p/k* in roots. (**A**) Different expressed *MaSPL* gene identified By RNA sequencing. (**B**) Expression profiling of known *miR156* members and their subgroups based on sRNAome. Ck: watering + non- inoculation; T1: drought + non- inoculation; T2: drought + inoculation.

**Figure 8:**
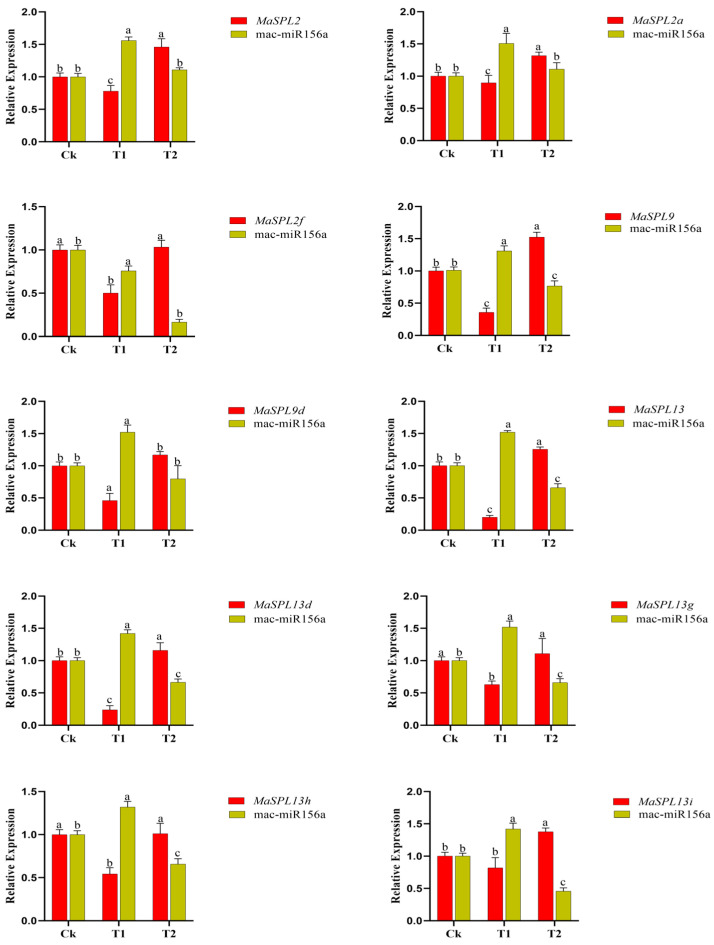
Relative expression profiles of *MaSPL* genes under drought (T1) and drought + inoculation (T2) treatments compared with the control (Ck), as determined by qRT-PCR. Values represent the mean of three biological replicates (*n* = 3) and are presented as fold changes calculated using the 2^−ΔΔCT^ method relative to the control. Error bars indicate the standard deviation (SD). Different lowercase letters above the bars denote significant differences among treatments at *p* < 0.05 according to Tukey’s HSD test.

Validation of RNA-seq expression and miRNA expression patterns from roots (Supplementary Tables S3 and S4, Figure 7A,B) by qRT-PCR confirmed that drought stress and fungal inoculation differentially regulate *MaSPL* family members in a manner consistent with *miR156a*-mediated modulation (Figure 8). Under drought stress (T1), all validated *MaSPL* genes showed reduced expression compared to control, with *MaSPL13* exhibiting the most pronounced suppression, followed by *MaSPL13d*, *MaSPL9*, *MaSPL9d*, and *MaSPL2.* Concurrently, *miR156a* expression was significantly elevated across all samples under drought stress, establishing a clear inverse correlation with *MaSPL* transcript levels.

In T2, it was demonstrated that fungal inoculation effectively rescues *MaSPL* expression from drought-induced suppression. *MaSPL13* showed the highest recovery, followed by *MaSPL13d, MaSPL2, MaSPL9, MaSPL9d*, and *MaSPL2f*. Critically, *miR156a* expression returned to near-baseline levels and showed downregulation under this condition, re-establishing the inverse relationship observed under drought stress.

These results support our hypothesis that drought stress suppresses *MaSPL* expression through *miR156a* induction, while fungal inoculation alleviates this suppression by reducing *miR156a* levels and directly activating SPL transcription. The clear inverse relationship between miR156a and *MaSPL* expression under different treatments, especially during drought and drought-plus-fungal exposure, strongly suggests that *miR156a* regulates SPL genes at the post-transcriptional level in response to abiotic stress as well as beneficial fungal association.

### 3.8. Determination of Antioxidant Enzyme Activities

**Figure 9:**
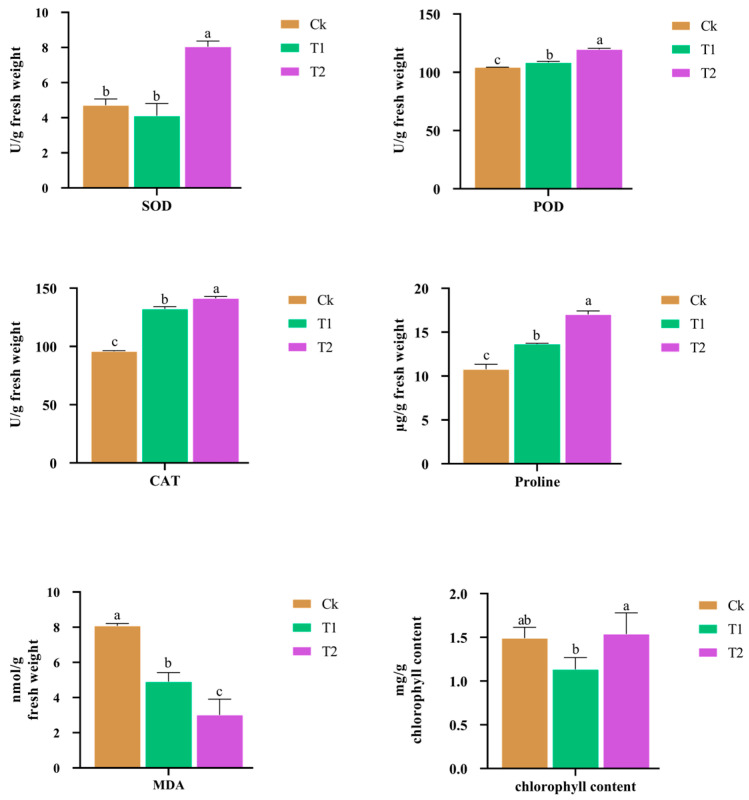
Effects of drought stress and *S. Indica* inoculation on antioxidant enzyme activities and physiological traits in banana. Superoxide dismutase (SOD) activity, peroxidase (POD) activity, catalase (CAT) activity, malondialdehyde (MDA) content, proline content, and chlorophyll content under different treatments: Data are presented as mean ± SD. Different letters above the bars indicate significant differences among treatments at *p* < 0.05.

Drought stress with *S. indica* inoculation significantly influenced antioxidant enzyme activities, osmotic balance, membrane stability, and chlorophyll content (Figure 9). SOD activity was highest in T2, while Ck and T1 showed no significant difference. POD and CAT activities increased under drought and were further enhanced by *S. indica* inoculation. Proline content showed a similar trend, with the highest accumulation observed in T2. MDA content decreased in drought-treated plants and reached its lowest level in T2, indicating less membrane damage under fungal inoculation. Chlorophyll content was reduced in T1 but was maintained in T2 at a level comparable to the control. These findings suggest that *S. indica* improves drought tolerance by strengthening antioxidant defense, enhancing osmotic adjustment, reducing membrane damage, and preserving photosynthetic capacity.

## 4. Discussion

We identified 38 MaSPL genes, all of which contained the canonical SBP domain and were predicted to localize to the nucleus. The encoded proteins varied in length from 171 to 506 amino acids, reflecting considerable diversity that could be linked to gene duplication events or genome complexity. The molecular weights of these proteins ranged from 18.981 to 56.488 kDa, and their pI values ranged from 5.84 to 9.67. These results indicate that most MaSPL proteins are alkaline, with an average pI of 8.54. According to the instability index, all MaSPLs were predicted to be unstable because their values were greater than 40. Moreover, the negative GRAVY values suggest that all proteins are hydrophilic. The subcellular localization prediction revealed that they were localized in the nucleus.

Based on the phylogenetic, comparison with other plant crops reveals a diverse gene distribution including 17 from *A. thaliana* [10], and 18 from *O. sativa* [13]. The tree is divided in VI clade, but no member of *MaSPL* was found in clades IV and VI. This suggests that diversification of the SPL gene family likely preceded the evolutionary divergence of monocots and dicots. Other monocots (*C. quinoa*, *H. vulgare*) and rice also lacked subfamily IV SPLs [53,54]. *AtSPL6* gene was reported to play a role in defense responses in *Arabidopsis*. In our phylogenetic analysis, *AtSPL6* gene showed a close relationship with *MaSPL13* members, suggesting that these genes may have related functional roles. Moreover, *SPL13* genes in other crops have been reported to be involved in drought tolerance. Compared with other monocots, including orchids and rice that retain subfamily VI members, most species harbor only a single SPL gene within this subfamily [55,56]. This pattern suggests that subfamily VI members may have limited functional significance and could be evolutionarily less conserved in banana. In contrast, clades I and II constitute the largest groups within the *MaSPL* family, a distribution pattern that is consistent with that observed in *Arabidopsis*. Genes grouped within the same phylogenetic clades also displayed similar motif composition and exon–intron organization, pointing to a relatively stable structural framework within the family. A comparable pattern has been reported in other species, including pecan [57], *Eucalyptus grandis* [58], and alfalfa [59], where SPL members within the same clades retain similar domain organization and gene structure despite expansion of the family across the genome.

Introns play a significant role in species evolution by increasing gene length, facilitating recombination, and contributing to gene regulation [60]. Earlier studies have reported that genes intricate in rapid stress responses frequently lack introns, since intronless genes can be transcribed more efficiently and may therefore enable faster regulatory responses during growth and development [61]. In the present study, the exon–intron organization, conserved motifs, and domain architecture of the 38 identified *MaSPL* genes were systematically analyzed (Figure 2). All *MaSPL* members possessed the conserved SBP domain, a characteristic feature of the SBP superfamily. The number of exons reached from two to six among the identified genes. Subfamilies I and II exhibited relatively complex gene structures, with a greater abundance of intron and exon regions. Notably, *MaSPL2f* contained six exons in clade I, whereas *MaSPL8c* in clade II, together with *MaSPL5, MaSPL4*, and *MaSPL4a* in clade I, contained only two exons. Analysis of conserved protein motifs further showed that most *MaSPL* proteins within the same subfamily shared similar motif compositions, whereas the arrangement and distribution of motifs differed among subfamilies. In contrast, *MaSPL13a*, *MaSPL13b*, *MaSPL13e*, and *MaSPL13d* lacked motif V. For example, motif 1 and motif 2 were identified in all *MaSPL* proteins. These findings are similar to *Hordeum vulgare* L. [53]. It indicates that *MaSPL* proteins may have undergone functional diversification.

Chromosomal distribution showed uneven distribution on ten chromosomes (02–11) (Figure 3A). The predominance of segmental duplication and the consistently low Ka/Ks ratios observed here further suggest that most duplicated *MaSPL* genes have been retained under purifying selection. Taken together, these results support the view that the banana SPL family has preserved core regulatory features during evolution while allowing divergence in expression behavior and, potentially, biological function.

Promoter analysis offers valuable insight into the regulatory networks and response mechanisms underlying gene expression. *Cis*-acting element prediction in the promoter regions of *MaSPL* genes revealed three major functional categories, namely light-responsive, hormone-responsive, and biotic/abiotic stress-responsive elements. These promoter regions were enriched with multiple *cis*-elements related to light response, phytohormone signaling, and stress regulation. In particular, the abundance of ABRE-, MYB/MYC-, and ERF-associated *cis*-elements indicates that *MaSPL* genes are likely involved in ABA-mediated signaling, dehydration responses, oxidative stress adaptation, and broader hormone-dependent regulatory processes. This interpretation is compatible with reports from other SPL systems in which promoter architecture suggests integration of developmental and stress-related cues [58]. At the same time, SPL genes do not appear to play uniform roles under abiotic stress. In rice, *OsSPL10* was shown to regulate drought tolerance through effects on ROS homeostasis, stomatal behavior, and downstream expression of *OsNAC2*, indicating that individual SPL members can act as important components of stress-responsive regulatory networks [62]. The strong repression of genes related to the *MaSPL2, MaSPL9*, and *MaSPL13* groups in the present study therefore most likely reflects functional specialization within the banana SPL family rather than a single common role for all SPL genes during drought.

A previous study [37] reported 56 putative SPL genes in *M. acuminata* and mainly examined their possible roles in fruit development and ripening. In the present study, ten *MaSPL* genes were predicted as potential targets of miR156, mainly belonging to the *MaSPL2, MaSPL9*, and *MaSPL13* subfamilies, which correspond to SPL members previously reported in [37]. Unlike the developmental focus of that study, our results indicate that these miR156-targeted *MaSPL* genes are transcriptionally responsive to drought stress and are further modulated by *Serendipita indica* inoculation. The expression data support the presence of a drought-responsive miR156–SPL regulatory module in banana: under drought, *MaSPL13*, *MaSPL13d*, *MaSPL9*, *MaSPL9d*, and *MaSPL2*, while *miR156a* was strongly induced, confirming a clear inverse relationship between *miR156a* and its target *MaSPL*. Under fungal inoculation, the expression of these *MaSPL* targets was largely restored and *miR156a* declined toward basal levels, suggesting that *S. indica* alleviates drought-induced repression of the *miR156a–MaSPL* module.

This inverse pattern is consistent with studies in other systems showing that stress-induced accumulation of *miR156a* reduces the abundance of selected SPL transcripts and contributes to stress adaptation. In alfalfa, *miR156a* overexpression improved drought tolerance, at least in part through repression of *SPL13*, accompanied by reduced water loss, improved stomatal conductance, and stronger protective responses under water deficit [63]. A similar relationship was described in apple, where the *miR156ab–SPL13* module enhanced drought tolerance by regulating auxin metabolism and antioxidant enzyme activity [64]. In light of these findings, the drought-induced increase in *miR156a* observed here likely contributes to the suppression of selected *MaSPL* genes as part of the regulatory shift from growth-related transcriptional programs toward stress adjustment.

*S. indica* substantially altered the drought-responsive behavior of the banana miR156–SPL system. Inoculation alone increased the expression of most tested *MaSPL* genes, and under combined drought and inoculation treatment it alleviated much of the drought-induced repression while reducing miR156a expression toward basal levels. This pattern suggests that fungal colonization acts upstream of the miR156–SPL node by changing the physiological conditions that normally favor miR156a accumulation during drought. Previous work supports such an interpretation. In trifoliate orange, *S. indica* improved drought tolerance by reducing oxidative damage and modifying antioxidant defense and fatty-acid composition [65], while in white clover, *S. indica* accelerated the ascorbate–glutathione cycle and improved ROS scavenging under water stress [66]. Recent work in tea also showed that *S. indica* alleviated drought injury through changes in osmotic regulation, antioxidant protection, transcriptional regulation, and flavonoid biosynthesis [67]. These studies do not prove the same mechanism in banana, but they make the present expression pattern biologically credible. A reasonable interpretation is that fungal colonization lowers drought-associated stress intensity, thereby weakening miR156a induction and permitting recovery of *MaSPL* transcript accumulation.

A systematic review of water stress in *M. acuminata* concluded that integrated molecular studies of drought responses in banana remain limited and that further work is needed on regulatory mechanisms underlying stress adaptation [68]. Within that broader gap, the present study extends banana SPL biology beyond developmental and postharvest contexts into drought–microbe interaction. This is also consistent with recent evidence that *miR156a*-targeted SPLs in banana are involved in stress-related regulation beyond development. In banana fruit, the *miR156c–SPL4* module has been shown to participate in chilling responses through downstream regulation of the *miR528–MaPPO* pathway and related oxidative processes [69]. Notably, the *SPL4* gene described in that study may not directly correspond to the *MaSPL4* gene identified in the present study due to differences in gene annotation and naming conventions; therefore, this comparison is based on functional similarity rather than strict gene identity. Although chilling stress is distinct from drought, that study strengthens the view that *miR156a*-targeted SPL in banana are responsive regulatory components in environmental stress signaling. The present results now suggest that a related regulatory logic may also operate during drought, particularly in the *MaSPL2, MaSPL9*, and *MaSPL13* groups.

The study establishes genome-wide identification, promoter analysis, target prediction, and expression profiling, but it does not yet provide direct functional evidence for individual *MaSPL* genes. Likewise, *miR156a* targeting was inferred from bioinformatics prediction and inverse expression patterns rather than validated by cleavage assays or reporter analysis. The proposed role of *S. indica* in modulating the miR156–SPL module therefore remains an interpretation based on transcriptional behavior rather than a demonstrated mechanism. No direct measurements of ABA content, ROS dynamics, stomatal regulation, or downstream SPL targets were included in the present work. These limitations do not weaken the value of the dataset, but they do define the next steps clearly. Functional validation of key *MaSPL* genes, experimental confirmation of *miR156a*-target relationships, and combined physiological and molecular analysis under drought with and without fungal colonization will be needed to resolve how this regulatory system operates in banana. The results support a model in which drought stress is associated with induction of *miR156a* and repression of selected *MaSPL* genes, whereas *S. indica* partially reverses this transcriptional pattern. The banana SPL family appears to be evolutionarily conserved yet environmentally responsive, and the present findings identify a small set of candidate genes for future work on drought adaptation and endophyte-mediated stress regulation.

## 5. Conclusions

In conclusion, our findings demonstrate that the banana SPL gene family represents a conserved yet stress-responsive regulatory network with an important role in drought adaptation. The inverse expression pattern of *miR156a* and several *MaSPL* genes suggests that drought-induced repression of *MaSPL* is mediated, at least in part, through the *miR156–SPL* regulatory module, while *Serendipita indica* appears to alleviate this repression by modulating upstream stress-related signaling. Together, these results provide a mechanistic framework linking SPL regulation, drought response, and endophyte-mediated stress mitigation in banana, and they identify key *MaSPL* candidates for future functional validation and crop improvement.

## Figures and Tables

**Table 1:** Genomic characterization of the *MaSPL* gene family in banana.

Gene Id	Id Rename	Chr	Amino Acid	Start Point	End Point	Strand	Exon	Intron	Subcellular Localization
Macma4_02_g06500.1	*MaSPL4*	chr02	215	21555007	21556402	+	2	1	nucleus
Macma4_02_g09590.1	*MaSPL13*	chr02	393	23774152	23777264	+	3	2	nucleus
Macma4_03_g05540.1	*MaSPL2*	chr03	464	3639311	3644326	−	4	3	nucleus
Macma4_03_g11410.1	*MaSPL9*	chr03	364	8479309	8485386	−	3	2	nucleus
Macma4_03_g18590.1	*MaSPL13a*	chr03	350	31240143	31242298	−	3	2	nucleus
Macma4_03_g30390.1	*MaSPL2a*	chr03	469	40618463	40623496	+	4	3	nucleus
Macma4_04_g01510.1	*MaSPL9a*	chr04	368	1422155	1426098	+	3	2	nucleus
Macma4_04_g04150.1	*MaSPL9b*	chr04	307	3342931	3344149	+	3	2	nucleus
Macma4_04_g05990.1	*MaSPL2b*	chr04	471	4581898	4586821	+	5	4	nucleus
Macma4_04_g13010.1	*MaSPL2c*	chr04	423	9489145	9494926	+	4	3	nucleus
Macma4_04_g18310.1	*MaSPL13b*	chr04	339	23350200	23351766	−	3	2	nucleus
Macma4_04_g29160.1	*MaSPL8*	chr04	356	36231423	36233186	+	3	2	nucleus
Macma4_04_g32230.1	*MaSPL5*	chr04	187	38649976	38650826	+	2	1	nucleus
Macma4_04_g35760.1	*MaSPL10*	chr04	347	40928542	40929928	+	3	2	nucleus
Macma4_04_g42180.1	*MaSPL4a*	chr04	171	44764114	44765956	+	2	1	nucleus
Macma4_05_g13770.1	*MaSPL10a*	chr05	355	9981841	9985666	+	3	2	nucleus
Macma4_05_g25410.1	*MaSPL2d*	chr05	494	41124138	41135584	−	5	4	nucleus
Macma4_05_g26050.1	*MaSPL2e*	chr05	456	41674593	41679614	+	5	4	nucleus
Macma4_05_g28440.1	*MaSPL13c*	chr05	391	43271978	43275549	−	3	2	nucleus
Macma4_06_g07880.1	*MaSPL13d*	chr06	369	5592497	5601851	+	3	2	nucleus
Macma4_06_g30570.1	*MaSPL13e*	chr06	273	35955998	35957388	−	3	2	nucleus
Macma4_06_g39570.1	*MaSPL2f*	chr06	506	42212601	42222131	−	6	5	nucleus
Macma4_07_g02010.1	*MaSPL9c*	chr07	377	1570316	1575635	+	3	2	nucleus
Macma4_07_g02800.1	*MaSPL13f*	chr07	239	2190144	2191397	+	3	2	nucleus
Macma4_07_g05150.1	*MaSPL8a*	chr07	343	3753042	3754527	−	3	2	nucleus
Macma4_07_g23410.1	*MaSPL9d*	chr07	363	34327242	34330591	−	3	2	nucleus
Macma4_07_g25950.1	*MaSPL13g*	chr07	400	36080600	36085680	−	3	2	nucleus
Macma4_08_g17020.1	*MaSPL13h*	chr08	401	26566241	26572127	−	3	2	nucleus
Macma4_08_g27110.1	*MaSPL9e*	chr08	367	45193678	45200876	+	3	2	nucleus
Macma4_09_g14330.1	*MaSPL2g*	chr09	458	9769241	9775951	−	4	3	nucleus
Macma4_09_g19220.1	*MaSPL9f*	chr09	356	15082047	15089551	−	3	2	nucleus
Macma4_09_g21270.1	*MaSPL8b*	chr09	372	32125223	32127429	−	3	2	nucleus
Macma4_09_g24190.1	*MaSPL9g*	chr09	343	40606548	40608342	+	3	2	nucleus
Macma4_09_g25000.1	*MaSPL2h*	chr09	440	41317217	41322303	−	4	3	nucleus
Macma4_09_g29910.1	*MaSPL9h*	chr09	369	45359810	45363469	+	3	2	nucleus
Macma4_10_g06830.1	*MaSPL2i*	chr10	473	13521635	13530534	+	4	3	nucleus
Macma4_10_g11050.1	*MaSPL8c*	chr10	228	24333643	24334715	+	2	1	nucleus
Macma4_11_g18990.1	*MaSPL13i*	chr11	346	29821305	29824782	+	4	3	nucleus

+, forward strand; –, reverse strand.

**Table 2:** Physicochemical properties of genome-wide identified *MaSPL* family gene members in banana.

Gene Id	Id Rename	Molecular Weight (KDa)	Theoretical pI	Instability Index	Aliphatic Index	GRAVY
Macma4_02_g06500.1	*MaSPL4*	23.746	9.24	71.95	52.74	−0.877
Macma4_02_g09590.1	*MaSPL13*	42.562	6.5	63.46	63.82	−0.48
Macma4_03_g05540.1	*MaSPL2*	50.695	9.13	55.78	63.32	−0.581
Macma4_03_g11410.1	*MaSPL9*	38.400	8.94	59.48	50.82	−0.543
Macma4_03_g18590.1	*MaSPL13a*	37.979	5.84	59.59	64.4	−0.476
Macma4_03_g30390.1	*MaSPL2a*	51.391	8.63	55.5	64.33	−0.626
Macma4_04_g01510.1	*MaSPL9a*	38.962	9.26	57.05	56.01	−0.491
Macma4_04_g04150.1	*MaSPL9b*	33.463	9.16	59.2	62.57	−0.453
Macma4_04_g05990.1	*MaSPL2b*	51.599	9.45	51.78	56.45	−0.684
Macma4_04_g13010.1	*MaSPL2c*	46.692	9.14	52.78	65.56	−0.667
Macma4_04_g18310.1	*MaSPL13b*	36.979	8.39	57.19	54.99	−0.711
Macma4_04_g29160.1	*MaSPL8*	39.558	6.9	69.19	52.64	−0.777
Macma4_04_g32230.1	*MaSPL5*	20.709	9.67	81.59	39.2	−1.226
Macma4_04_g35760.1	*MaSPL10*	38.175	9.02	53.31	70.95	−0.409
Macma4_04_g42180.1	*MaSPL4a*	18.981	9.48	78.42	37.78	−1.348
Macma4_05_g13770.1	*MaSPL10a*	38.828	8.84	65.88	65.49	−0.537
Macma4_05_g25410.1	*MaSPL2d*	54.705	9.14	42.13	63.54	−0.63
Macma4_05_g26050.1	*MaSPL2e*	49.860	9.1	52.31	67.52	−0.352
Macma4_05_g28440.1	*MaSPL13c*	42.337	6.69	57.64	61.1	−0.531
Macma4_06_g07880.1	*MaSPL13d*	39.953	7.65	62.49	67.64	−0.451
Macma4_06_g30570.1	*MaSPL13e*	29.960	6.79	61.08	63.92	−0.517
Macma4_06_g39570.1	*MaSPL2f*	56.488	9.44	54.99	63.97	−0.625
Macma4_07_g02010.1	*MaSPL9c*	40.223	9.07	56.82	60.27	−0.452
Macma4_07_g02800.1	*MaSPL13f*	26.741	6.5	61.79	69.33	−0.564
Macma4_07_g05150.1	*MaSPL8a*	37.747	9.61	64.05	53.21	−0.69
Macma4_07_g23410.1	*MaSPL9d*	38.547	9.5	59.59	54.1	−0.583
Macma4_07_g25950.1	*MaSPL13g*	43.381	8.22	58.03	64.07	−0.524
Macma4_08_g17020.1	*MaSPL13h*	44.054	6.9	54.67	63.42	−0.492
Macma4_08_g27110.1	*MaSPL9e*	39.081	8.64	48.24	46.89	−0.636
Macma4_09_g14330.1	*MaSPL2g*	50.116	9.14	58.15	65.61	−0.562
Macma4_09_g19220.1	*MaSPL9f*	37.801	9.01	55.36	51.04	−0.568
Macma4_09_g21270.1	*MaSPL8b*	41.044	8.56	63.92	63.31	−0.558
Macma4_09_g24190.1	*MaSPL9g*	36.833	8.68	54.26	62.59	−0.459
Macma4_09_g25000.1	*MaSPL2h*	48.559	8.06	52.04	60.18	−0.68
Macma4_09_g29910.1	*MaSPL9h*	39.364	8.48	49.74	56.07	−0.518
Macma4_10_g06830.1	*MaSPL2i*	52.071	9.15	52.51	70.57	−0.523
Macma4_10_g11050.1	*MaSPL8c*	25.634	9.47	66.55	60.35	−0.662
Macma4_11_g18990.1	*MaSPL13i*	38.391	9.39	57.4	66.18	−0.599

## Data Availability

All relevant data is available within the manuscript and Supplementary Materials.

## Supplementary Materials

The following supporting information can be downloaded at: https://www.mdpi.com/article/10.3390/plants15091386/s1, Supplementary Figure S1: Multiple sequence alignment and conservation analysis of the SBP domain in MaSPL and AtSPL proteins; Supplementary Figure S2; Exon/intron distribution analysis of *AtSPL* gene members in *Arabidopsis thaliana*, to compare with banana; Supplementary Figure S3: Transcription Factor Binding Site Analysis of *MaSPL* Promoters; Supplementary Table S1: Gene name and gene ID of SPLs in Arabidopsis and rice; Supplementary Table S2: The length and consensus sequence of 10 motifs identified in *MaSPL*; Supplementary Table S3: Log2fc data table of *MaSPL* during different treatment; Supplementary Table S4: Log2fc data table of known miRNA during treatment; Supplementary Table S5: *Cis*-acting element in the *MaSPL* promoter region; Supplementary Table S6: Summary of primers used in qRT-PCR; Supplementary Table S7: Raw Ct values of mRNA and miRNA; Supplementary Table S8: Protein sequences of 38 *MaSPL*.

## Abbreviations

The following abbreviations are used in this paper:

*Ma*	*Musa acuminata*
*At*	*Arabidopsis thaliana*
*Os*	*Oryza sativa*
*S. indica*	*Serendipita indica*
*P. indica*	*Piriformospora indica*
PDA	Potato Dextrose Agar
PDB	Potato Dextrose broth
FWC	Field water capacity
SPL	SQUAMOSA promoter-binding protein-like
miRNAs	MicroRNAs
mRNA	messenger RNA
TFs	transcription factors
PI	isoelectric point
II	instability index
MW	molecular weight
qRT-PCR	real-time reverse transcription PCR
ROS	reactive oxygen species
ABA	abscisic acid
GRAVY	grand average of hydropathicity
