# Supplementary material for: Genome-Wide Identification and Expression Profiling of the SPL Gene Family in Musa acuminata: Insights into Their Response to Drought Stress and Serendipita indica Inoculation
Source: Plants (Basel). 2026 Apr 30;15(9):1386. doi: 10.3390/plants15091386 (PMC13165380; doi:10.3390/plants15091386)
Supplement: Supplementary file 1 [file plants-15-01386-s001.zip › plants-4252222-Suplementray figures.pdf]

Supplementary Files

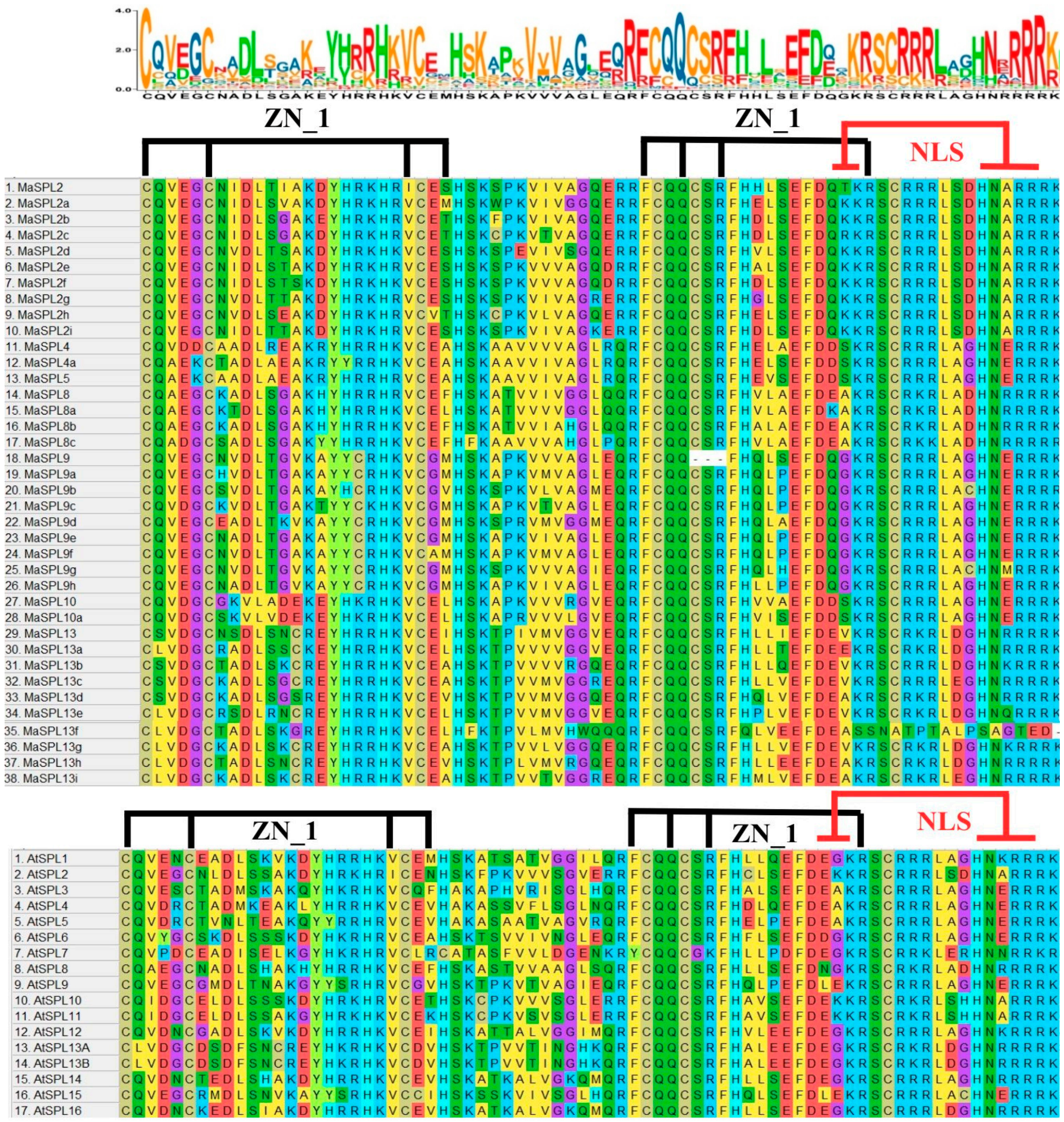

Figure S1. Multiple sequence alignment and conservation analysis of the SBP domain in MaSPL and AtSPL proteins. The alignment highlights the highly conserved SBP domain (~76 amino acids) across Musa acuminata (top panel) and Arabidopsis thaliana (bottom panel). A sequence logo at the top illustrates the degree of amino acid conservation. Structural motifs are annotated above and below the sequences, identifying two zinc-finger motifs (labeled ZN_1, corresponding to C3H and C2HC types) and a bipartite nuclear localization signal (NLS) located toward the C-terminal region. The color-coded residues indicate chemical properties, demonstrating the canonical domain organization required for nuclear DNA binding.

Figure S2; Exon/intron distribution analysis of *AtSPL* gene members in *Arabidopsis thaliana*

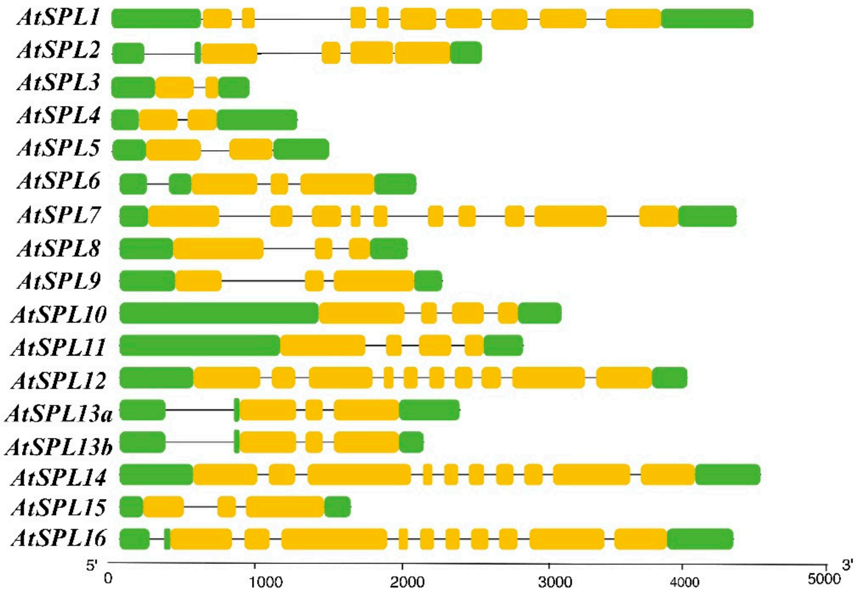

Figure S2. Exon/intron distribution analysis of *AtSPL* gene members in *Arabidopsis thaliana,* to compare with banana

Figure S3; **Transcription Factor Binding Site Analysis of *MaSPL* Promoters**

To better understand the potential regulatory framework of the MaSPL gene family, the distribution of transcription factor (TF) associations across all MaSPL members was analyzed and visualized using hierarchical clustering. The results revealed a highly diverse TF interaction landscape, indicating that MaSPL genes may be regulated through multiple transcriptional pathways.

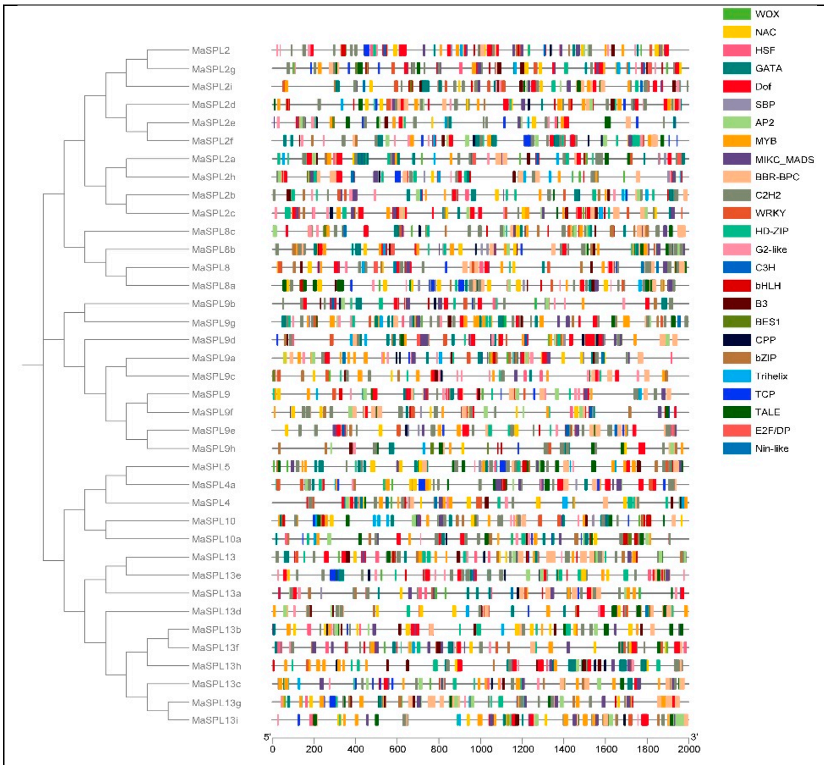

Figure S3. Transcription Factor Binding Site Analysis of *MaSPL* Promoters
